# Primary Cardiac Angiosarcoma: A Fatal Disease

**DOI:** 10.1155/2009/591512

**Published:** 2009-08-20

**Authors:** L. Antonuzzo, V. Rotella, F. Mazzoni, L. Doni, D. Bianchini, F. Garbini, V. Maio, F. Di Costanzo

**Affiliations:** ^1^Oncologia Medica, Azienda Ospedale Universitaria Careggi, Viale Pieraccini 17, 50139 Firenze, Italy; ^2^Dipartimento di Patologia Umana ed Oncologia, Azienda Ospedale Universitaria Careggi, Viale Pieraccini 17, 50139 Firenze, Italy

## Abstract

A 42-year-old man with a cardiac tamponade underwent an urgent pericardiotomy that showed tumoral tissue, covering the surface of the right atrium. The tumor was then partially excised, and the histological examination revealed the presence of a moderately-differentiated angiosarcoma. The patient was then referred to the oncology unit and scheduled for a chemotherapy schedule including Epirubicin (60 mg/m^2^, on days 1 and 2) plus Ifosfamide (2000 mg/m^2^, on days 1 to 3) and Uromitexan (2000 mg/m^2^ at hours 0, 4, 8 after IFO). All drugs were administered every three weeks. After two cycles, a restaging work-up revealed a partial remission. The treatment was continued for another two cycles. A new evaluation by cardiac MRI evidenced a local and distant (lung) progression of disease. The patient died after three months. This paper confirms that cardiac angiosarcoma is a fatal disease, and the prognosis is usually 6–11 months from time of diagnosis.

## 1. Case Report

**Figure 1:**
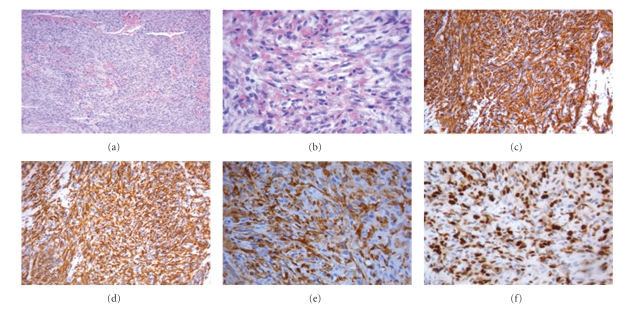
(a) (colouring H&E 10×) Angiosarcoma moderately differentiated. (b) (colouring H&E 40×) Greater enlargement angiosarcoma moderately differentiated. (c) (20×) Immunohistochemical investigation tumor cells were positive for CD 31. (d) (20×) Immunohistochemical investigation tumor cells were positive for CD34. (e) (20×) Immunohistochemical investigation tumor cells were positive for FVIII rAg. (f) (40×) Positive for Mib-1 60%.

**Figure 2:**
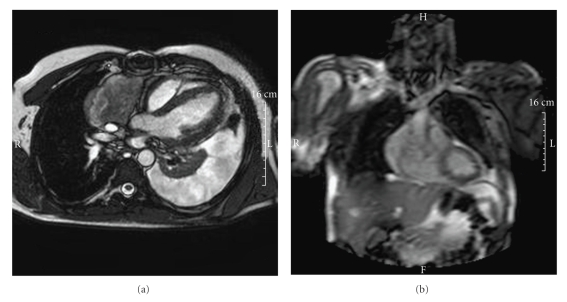
Voluminous left pleural effusion. Large mass extending from the free wall of the right atrium to the anterior mediastinum beside the superior vena cava and ascending aorta and above until the right pulmonary artery.

A 42-year-old man with a cardiac tamponade was referred to the Department of Cardiovascular Surgery of our hospital to be submitted to an urgent pericardiotomy following a median sternotomy. His medical history dated back to July 2006 when he was admitted to the Cardiological Department of General Hospital in a suburb of Florence, complaining of a three-week history of chest pain and dyspnea. On admission, the patient was in good general condition. Blood pressure (BP) and pulse were normal. Heart auscultation was unremarkable except for a faint pericardial rub; chest examination showed normal breath sounds. Blood tests showed hemoglobin 11.5 g/dl, leukocyte count 7000/mm^3^, platelet count 250 × 10^3^/mm^3^; liver and renal biochemistry, clotting profile, and erythrocyte sedimentation rate (ESR) were normal. At this time chest X-ray showed a mild cardiomegaly. An electrocardiogram (EKG) showed normal sinus rhythm without acute ischemic alterations. Subsequently, he was submitted to transthoracic echocardiography (TTE) that documented a voluminous pericardial effusion with normal left ventricular function. The patient was treated with pericardiocentesis, and a total of 1500 cc of hemorrhagic pericardial fluid was aspirated. Laboratory and cytological analysis of the pericardial fluid resulted negative for malignancy. After a complete diagnostic work-up he was diagnosed as having acute pericarditis (of likely viral origin) and he was discharged in good condition from the hospital for follow-up and ambulatory investigation. A month after discharge, the patient returned to the emergency room of our hospital with a second episode of chest pain, rapidly progressive dyspnea, hypotension, and tachycardia. The TTE confirmed the clinical suspect of recurrent pericardial effusion complicated by a cardiac tamponade. Then it was necessary to again perform an emergency pericardiocentesis with aspiration of 70 cc alone of hemorrhagic pericardial fluid and to refer the patient to the Department of Cardiovascular Surgery. The patient underwent an urgent operation consisting in a median sternotomy and longitudinal pericardiotomy. During the operation a tumoral tissue was found covering the surface of the right atrium and most of the anterolateral pericardium. The tumor was then partially excised. The histological examination revealed the presence of a moderately differentiated angiosarcoma. The histological features of the neoplasm were quite variable among the different zones of the lesion. At low power, the tumor showed an alternation of hyper- and hypocellular areas with the presence of dense keloid-type collagen alternating to myxoid areas (Figure 1(a)). Due to these features, on frozen section examination, the hypothesis of solitary fibrous tumor was raised. In areas, the tumor was virtually avascular and composed of compact masses of moderately pleomorphic spindled and epithelioid cells within a collagenous stroma. In other areas, the neoplasm showed clusters of small and short capillary-like slits, sometimes in stellate configuration, lined with variably atypical tumor cells (Figure 1(b)). In other portions, the tumor displayed large, well-formed, but irregularly contoured vascular spaces in continuity with small sinusoidal-type tributaries or a relatively pure sinusoidal vascular pattern, often intersected by broad collagenous bands. The immunohistochemical investigation documented that tumor cells were positive for CD31 (Figure 1(c)), CD34 (Figure 1(d)), Von Willebrand Factor (VWF) (Figure 1(e)), and about 60% of them were positive for Mib-1 (Figure 1(f)), whereas they were negative for EMA, bcl-2, smooth muscle actin, common muscle actin, desmin, and protein S-100. The patient was moved to the Department of Internal Medicine in our hospital. At that time, hemodynamic parameters were stable, chest X-ray showed cardiomegaly and left-sided pleural effusion and TTE detected only a small pericardial effusion with normal cardiac size and ventricular function (ejection fraction, evaluated with TTE, was 63%). A further evaluation with cardiac magnetic resonance imaging (MRI) showed a large mass (53 mm) extending from the free wall of the right atrium to the anterior mediastinum beside the superior vena cava and ascending aorta, above up to the right pulmonary artery, clearly revealed irregular signals in the mass after gadolinium enhancement (Figure 2). The extensive study using computed tomography (CT) of the brain, chest and abdomen and positron emission tomography (PET) showed no metastasis. The patient was then referred to our oncology unit and scheduled for a chemotherapeutic regimen including Epirubicin (EPI) (60 mg/m^2^, on days 1 and 2) plus Ifosfamide (IFO) (2000 mg/m^2^, on days 1 to 3) and Uromitexan (2000 mg/m^2^ at hours 0, 4, 8 after IFO). All drugs were administered every three weeks. After two cycles, a restaging work-up with CT plus PET and cardiac MRI revealed a partial response. The treatment was well tolerated and continued for another two cycles. A new evaluation by cardiac MRI and total body CT evidenced a local progression of disease and appearance of pulmonary metastases. The patient died after three months for occurrence of acute respiratory failure.

## 2. Discussion

Primary tumors of the heart are extremely rare, occurring at a frequency of 0.02% in autopsy series and the majority of them are benign [1]. Metastatic heart tumors are 20–40 times more frequent than primary cardiac tumors [2]. Statistically, heart cancer is listed with all cancers of the soft tissues. In a report of 12 487 consecutive autopsies, only seven cases of primary cardiac tumor were reported, most of which were benign [3]. Malignant tumors of the heart are most often sarcoma (76–78%) and angiosarcoma, the most aggressive histotype, accounts for about 31% of all primary cardiac malignant tumors [4–8]. There are also many other malignant subtypes including rhabdomyosarcoma, malignant fibrous histiocytoma, undifferentiated and fibrosarcoma [9]. Common age of presentation is the third to fifth decade and is more frequent in male (ratio male/female: 2-3/1). An angiosarcoma of the heart is considered primary if there is no evidence of previous or concomitant tumors in the soft tissue, bone, or subcutaneous tissue [10]. Angiosarcoma of the heart usually involves the right atrium, and it is characterized by a rapid and infiltrating growth within the myocardial wall, friability, and tendency toward bleeding [11]. In fact the clinical presentation with pericardial effusion and cardiac tamponade is frequent. Another life-threatening clinical presentation is the myocardial rupture due to tumor infiltration and necrosis of the wall [12]. Clinical presentation of the cardiac sarcoma is usually late and dependent on the effect of the local infiltration within the cardiac wall, the atrioventricular valves, the pericardium or the superior or inferior vena cava or from symptoms or signs secondary to the metastases.

The right atrium is the most common site of origin followed by the left atrium, right ventricle, and left ventricle. Unexplained chest pain, hemoptysis, clubbing, anemia, cardiomegaly, or metastatic manifestations may be the first clue to these rare yet devastating entities. Tachycardia, hypotension, pulsus paradoxus, and congestion of neck veins are hallmarks of pericardial effusion. Electrocardiogram may sometimes evidence arhythmia, conduction abnormalities, or low voltage complexes but the screening tool of choice for cardiac tumors is echocardiography which can detect an intracavitary mass, pericardial effusion, or a reduced pump function. Cardiac RMI or a transesophagael echocardiogram (TEE) is indicated to better define the exact location, the size, and the locoregional extension of cardiac neoplasm in absence of echocardiographic significant signs. TEE has a higher resolution (1–3 mm) than MRI (5–10 mm), while MRI is better to identify tissue composition with ability to differentiate solid, liquid, hemorrhagic and fatty structure. Pericardial fluid cytology is positive in 75–87% of patients. However, pericardial biopsies guided by pericardioscopy have a diagnostic value of 93.3–97%. A less invasive procedure is the transvenous endocardial biopsy but it often false-negative [13, 14]. Chest X-ray often evidence cardiomegaly, pulmonary congestion, or pericardial effusion. CT is useful not only to better define local extent of the tumor, but also to evidence possible metastasis quite frequent in angiosarcoma (80%) especially lung metastasis. The differential diagnosis is very broad and must include thrombus, vegetation, foreign body, intracardiac metastases, infectious and nonbacterial thrombotic or marantic endocarditis [15], coronary artery disease, constrictive cardiomyopathy, and bronchogenic carcinoma or mesothelioma [16]. Despite the availability of modern imagine techniques the diagnosis of cardiac sarcoma is usually late. The prognosis of cardiac angiosarcoma is poor with a median survival ranging from 6 to 11 months. The therapy for primary cardiac tumors is still controversial but the surgery represents the therapy of choice in the cases of localized disease and is conditioned by the site of tumor and by the frequent presence of metastases. The survival percentage in patients treated with surgery ranges from 2 to 55 months with a median survival of 14 months. Despite adverse prognostic data, there are reported cases of patients with angiosarcoma and treated with only partial resection followed by chemotherapy and radioteraphy who survived 34 or even 53 months [17, 18]. These long-term survivors were submitted to extensive surgical resection of the primary tumor, plus adjuvant multidisciplinary therapy and had absence of systemic disease at the time of diagnoses [18]. From these case reports we should consider chemotherapy and/or radiotherapy as adjuvant good options, after radical or debulking surgery, in order to improve the overall survival. In this case we used a combination of Ifosfamide and Epirubicin that is the most active treatment in sarcoma. Most frequently used in literature are the combinations of Cyclophosphamide, Doxorubicin, Vincristine, Dacarbazine (CyVADIC), and of Dacarbazine or Mitomycin-C, Doxorubicin, Cisplatin, and Vincristine [19]. Sinatra et al. reported that the combination of surgical resection and radiation can reduce the mass and eliminate symptoms despite an incomplete resection [20]. Radiation is limited to the concern for cardiotoxicity [21]. Kakizaki et al. reported a case of cardiac angiosarcoma treated with combination of chemotherapy and immunotherapy (Interleukin-2) with a survival of 30 months after surgery [22].

Cardiac transplant has been performed in some cases with early diagnosis and cardiac tumor not completely resectable but with worsened results because the immunosuppressive therapy can increase the risk of progression of cancer disease [23, 24].

In conclusion primary cardiac angiosarcoma is a fatal disease, and the prognosis is usually short (6–11 months); chemotherapy has a soft impact on prognosis [25].

## Abbreviations

BP: Blood pressure
ESR: Erythrocyte sedimentation rate
EKG: Electrocardiogram
TTE: Transthoracic echocardiography
VWF: Von Willebrand Factor
MRI: Magnetic resonance imaging
CT: Computed tomography
PET: Positron emission tomography
EPI: Epirubicin
IFO: Ifosfamide
TEE: Transesophageal echocardiogram.
